# Acetohydroxyacid synthase FgIlv2 and FgIlv6 are involved in BCAA biosynthesis, mycelial and conidial morphogenesis, and full virulence in *Fusarium graminearum*

**DOI:** 10.1038/srep16315

**Published:** 2015-11-10

**Authors:** Xin Liu, Qi Han, Jianhong Xu, Jian Wang, Jianrong Shi

**Affiliations:** 1 Institute of Food Quality and Safety, Jiangsu Academy of Agricultural Sciences, Jiangsu, China; 2 State Key Laboratory Breeding Base of Food Quality and Safety in Jiangsu Province, Jiangsu, China; 3 Key Laboratory of Control Technology and Standard for Agro-product Safety and Quality (Ministry of Agriculture), Jiangsu, China; 4 Key Laboratory of Agro-product Safety Risk Evaluation Nanjing (Ministry of Agriculture), Jiangsu, China; 5 Collaborative Innovation Center for Modern Grain Circulation and Safety, Jiangsu, China

## Abstract

In this study, we characterized FgIlv2 and FgIlv6, the catalytic and regulatory subunits of acetohydroxyacid synthase (AHAS) from the important wheat head scab fungus *Fusarium graminearum*. AHAS catalyzes the first common step in the parallel pathways toward branched-chain amino acids (BCAAs: isoleucine, leucine, valine) and is the inhibitory target of several commercialized herbicides. Both *FgILV2* and *FgILV6* deletion mutants were BCAA-auxotrophic and showed reduced aerial hyphal growth and red pigmentation when cultured on PDA plates. Conidial formation was completely blocked in the *FgILV2* deletion mutant ΔFgIlv2-4 and significantly reduced in the *FgILV6* deletion mutant ΔFgIlv6-12. The auxotrophs of ΔFgIlv2-4 and ΔFgIlv6-12 could be restored by exogenous addition of BCAAs but relied on the designated nitrogen source the medium contained. Deletion of *FgILV2* or *FgILV6* also leads to hypersensitivity to various cellular stresses and reduced deoxynivalenol production. ΔFgIlv2-4 lost virulence completely on flowering wheat heads, whereas ΔFgIlv6-12 could cause scab symptoms in the inoculated spikelet but lost its aggressiveness. Taken together, our study implies the potential value of antifungals targeting both FgIlv2 and FgIlv6 in *F. graminearum*.

Fusarium head blight (FHB), caused by pathogenic ascomycete fungi of the genus *Fusarium*, such as *Fusarium graminearum* (telemorph: *Gibberella zeae*), is a devastating disease in wheat, barley and other small grain cereals. As one of the most important cereal killers worldwide, FHB not only leads to great yield losses but also contaminates grains by producing mycotoxins that are hazardous to humans and livestocks^1^,^2^,^3^. FHB epidemics in Europe and North America have resulted in huge economic losses^4^ and in China FHB epidemics occur frequently in the central region of China, especially along the middle and lower reaches of the Yangtze River^5^, resulting in huge production losses to farmers and food safety concerns to the public.

Currently, resistant wheat cultivars for FHB are not available worldwide and fungicide application remains the most predominant approach for FHB management. The benzimidazole fungicide carbendazim targets beta-tubulin in fungi and has been extensively used in China for FHB management since the 1970 s. However, due to the long-term application, the fungus has developed high level drug resistance to carbendazim, especially in the wheat planting area along the middle and lower reaches of the Yangtze River^6^. Considering the current status of FHB control, alternative effective and low-toxic fungicides against *F. graminearum* are urgently needed.

Certain amino acid biosynthetic pathways which are highly conserved in fungi, yet are missing in mammals, are potential antifungal targets in the search for effective pesticides. The branched-chained amino acid (BCAA: isoleucine, Ile; leucine, Leu; Valine, Val) biosynthetic pathway is of particular interest as its conservation in fungi and absence in mammals makes it an appealing target for fungicide application as mammals should be immune to any detrimental effects of the dispersed fungicide. Currently, several classes of commercially used herbicides, including sulfonylureas, imidazolinones, and sulfonanilides, target this pathway in plants through the inhibition of acetohydroxyacid synthase (AHAS, EC2.2.1.6)^7^,^8^,^9^. AHAS is composed of the catalytic subunit encoded by *ILV2* and the regulatory subunit encoded by *ILV6* and catalyzes the first common step in BCAA biosynthesis for the conversion of pyruvate to 2-acetolactate in Val and Leu biosynthesis or 2-ketobutyrate to 2-aceto-2-hydroxybutyrate in Ile biosynthesis^10^,^11^. Recent studies have shown that inhibitors of AHAS, e.g. sulfonylureas, also are capable of inhibition in several different species of bacteria and fungi, including *Salmonella typhimurium, Burkholderia pseudomallei, Pseudomonas aeruginosa, Acinetobacter baumannii* and *Candida albicans*^12^,^13^,^14^,^15^,^16^, indicating a promising antimicrobial drug strategy targeting enzymes involved in the BCAA biosynthetic pathway.

Functional analysis of the *ILV2* gene encoding the catalytic subunit of AHAS has been carried out in several different species of fungi. In *Saccharomyces cerevisiae* and *Cryptococcus neoformans, ILV2* disruption mutants were viable in complete medium, however, the mutants lost viability rapidly and/or were avirulent during Ile and Val starvation (auxotrophic growth conditions)^17^,^18^. In *C. albicans*, an *ILV2* deletion mutant was also significantly attenuated in virulence^19^. More recently, Du *et al.* characterized *MgILV2* and *MgILV6* in the rice blast fungus *Magnaporthe oryzae*, and found that deletion mutants in both genes were BCAA-auxotrophic and defective in various infection-related phenotypes^20^.

In this study, we characterized the functions of *FgILV2* and *FgILV6* encoding the catalytic and regulatory subunits of AHAS by using a strategy of targeted gene deletion and gene complementation in the important wheat head scab fungus *F. graminearum* to investigate their possible roles in various cellular processes as well as the potential to serve as novel fungicide targets.

## Results

### Identification of the catalytic and regulatory subunits of AHAS in *F. graminearum*

Using the amino acid sequences of *S. cerevisiae* ScIlv6 (NP_009918.1) and ScIlv2 (NP_013826.1) as queries, homology searches in the *Fusarium graminearum* genome database (http://www.broadinstitute.org/annotation/genome/fusarium_group/MultiHome.html) revealed that the loci FGSG_06282.3 and FGSG_01086.3 (Broad accession number) share a high level of amino acid homology with ScIlv6 and ScIlv2, respectively, and were designated as FgIlv6 and FgIlv2. The regulatory subunit of AHAS FgIlv6 is predicted to contain an N-terminal ACT (aspartate kinase, chorismate mutase and TyrA) domain and a C-terminal AHAS small subunit. FGSG_06282.3 sequence (FgIlv6) exhibits high levels of amino acid homology to the corresponding Ilv6 from three other *Fusarium* species (*F. oxysporum* 93.27%, *F.verticillioides* 92.95%, *F. fujikuroi* 92.95%), as well as from *M. oryzea* (77.29%), *Aspergillus nidulans* (68.20%), and *S. cerevisiae* (59.31%) (Figure S1). FgIlv2 is predicted to contain three protein domains including a thiamine pyrophosphate enzyme (TPE) N-terminal binding domain, a TPE central domain, and a TPE C-terminal binding domain. Phylogenetic analysis of FgIlv2 and the corresponding Ilv2 from other species showed that unlike Ilv6, Ilv2 was less conserved among the four *Fusarium* species (*F. oxysporum* 74.75%, *F. verticillioides* 74.75%, *F. fujikuroi* 73.44%) (Figure S1).

*FgILV6* encodes 313 amino acids and contains only one predicted intron which is 50 bp in length and located from 759 to 808 bp of the nucleotides. *FgILV2* encodes 683 amino acids and contains seven predicted introns as listed: 124 bp in length located between nucleotides 117 and 240, 50 bp in length located between nucleotides 343 and 392, 77 bp in length located between nucleotides 461 and 537, 45 bp in length located between nucleotides 649 and 693, 55 bp in length located between nucleotides 1776 and 1830, 51 bp in length located between nucleotides 1898 and 1948, and 66 bp in length located between nucleotides 2390 and 2455. Sequencing of the 2049 bp cDNA of *FgILV2* and the 949 bp cDNA of *FgILV6* verified the existence of the predicted introns.

### Disruption and complementation of *FgILV2* and *FgILV6* genes in *F. graminearum*

Deletion mutants of *FgILV2* and *FgILV6* were successfully generated through PEG-mediated protoplast transformation of the purified DNA fragments of PCR products amplified with primer pair A1 + A4 or B1 + B4 using diluted plasmid pBS-ilv2-Del or pBS-ilv6-Del as templates with hygromycin as the selective agent. Complementation of the deletion mutants was conducted by transforming the full-length gene to the protoplasts of the corresponding deletion mutant using G418 sulfate as the selective agent. Correct integration of the transforming DNA fragments into the genome was verified with RT-PCR (Supplementary Figures S2B, S3B) and single transformation events confirmed by Southern blotting analysis (Figures S2C, S3C).

### Disruption of *F. graminearum FgILV2* and *FgILV6* genes results in auxotrophy, reduced aerial hyphal growth, and red pigmentation

**Figure 1:**
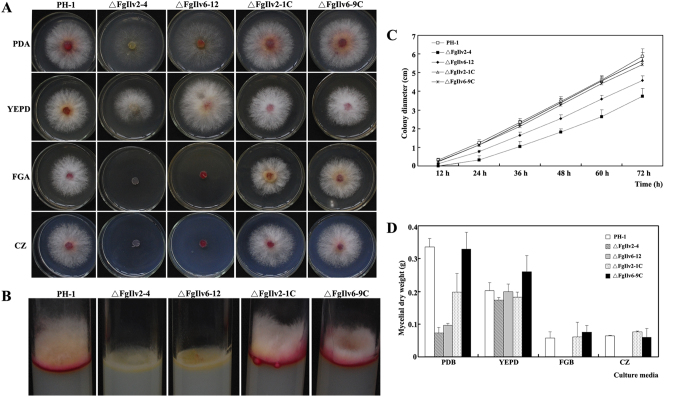
Deletion of the FgILV2 and FgILV6 genes led to reduced mycelial growth and red pigmentation. (**A**) Colony morphology of the wild-type strain PH-1, the *FgILV2* deletion mutant (ΔFgIlv2-4), the *FgILV6* deletion mutant (ΔFgIlv6-12), the *FgILV2* complemented strain (ΔFgIlv2-1C), and the *FgILV6* complemented strain (ΔFgIlv6-9C), on PDA, YEPD, FGA or CZ medium after 3 days of incubation at 25 °C. (**B**) Reduction of aerial hyphae production and red pigmentation of ΔFgIlv2-4 and ΔFgIlv6-12 on PDA medium after 3 days of incubation at 25 °C. (**C**) Reduced mycelial growth rate of ΔFgIlv2-4 and ΔFgIlv6-12 on PDA medium. (**D**) Reduced fungal biomass of ΔFgIlv2-4 and ΔFgIlv6-12 in liquid PDB, YEPD, FGB, and CZ after 2 days of incubation at 25 °C.

Previous studies have shown that *S. cerevisiae, C. albicans* and *M. oryzea ILV2* deletion mutants exhibited a starvation-cidal phenotype^17^,^18^,^19^,^20^. We therefore checked the growth of *FgILV2* and *FgILV6* deletion mutants on fructose gelatin agar (FGA) and czapek dox medium (CZ) containing no amino acids. As shown in Fig. 1A, the *FgILV2* deletion mutant ΔFgIlv2-4 and the *FgILV6* deletion mutant ΔFgIlv6-12 could not grow on FGA or CZ medium containing no amino acids. In contrast, when cultured on full-nutrition medium, the auxotrophy of ΔFgIlv2-4 and ΔFgIlv6-12 could be partially overcome on PDA medium despite the reduced aerial hyphae and fully restored on YEPD plates. When cultured on PDA plates, both ΔFgIlv2-4 and ΔFgIlv6-12 formed reduced aerial hyphae (Fig. 1A,B) and the growth rate of ΔFgIlv2-4 and ΔFgIlv6-12 was slower compared to the wild-type PH-1 (Fig. 1C). Results of the fungal biomass assays showed that the weight of dry mycelia harvested from PDB-grown cultures of both ΔFgIlv2-4 and ΔFgIlv6-12 was significantly reduced as compared to the wild-type PH-1 and the complemented strain. No visible difference was observed between the mutants and the wild-type when cultured in YEPD (Fig. 1D). In addition to the reduced aerial hyphal formation, both ΔFgIlv2-4 and ΔFgIlv6-12 exhibited reduced red pigmentation as shown in Fig. 1B. To further quantify this phenotype, relative expression levels of six genes (*PKS12, Gip1, Gip2, AurJ, AurF* and *AurR2*; see Table 1 for gene function description) involved in red pigmentation^21^,^22^,^23^ were detected by using quantitative realtime-PCR (qRT-PCR) and results showed that expression of all the six red pigment formation related genes was significantly down-regulated in both ΔFgIlv2-4 and ΔFgIlv6-12 (Table 1). Amino acid auxotrophy, mycelial growth defects and reduced red pigmentation of both deletion mutants could be restored by genetic complementation with the corresponding full-length gene.

### The *F. graminearum FgILV2* deletion mutant auxotroph is satisfied by addition of isoleucine and valine

**Figure 2:**
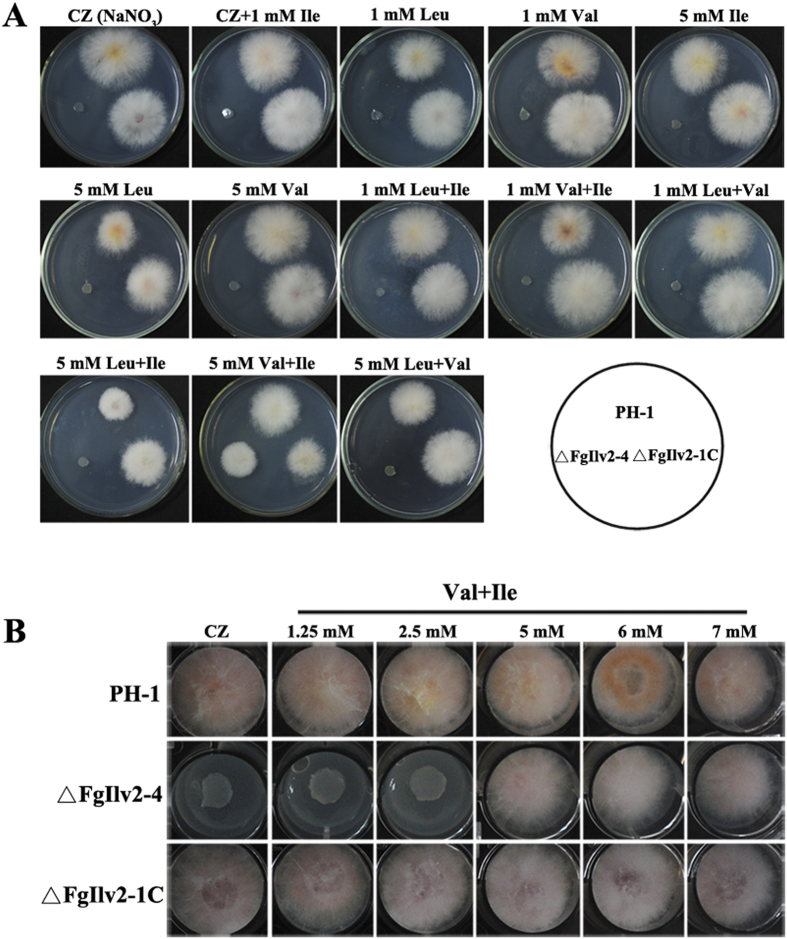
Exogenous addition of Val and Ile at 5 mM affect the growth of the auxotrophic mutant ΔFgIlv2-4 . (**A**) Mycelial morphology of PH-1, ΔFgIlv2-4 and ΔFgIlv2-1C cultured on CZ medium amended with amino acids at different concentrations indicated in the figure at 25 °C for 2 days. (**B**) Colony morphology of the indicated strains grown on CZ in the presence of 1.25, 2.5, 5, 6 and 7 mM Val and Ile at 25 °C for 2 days.

Growth of the wild-type PH-1, the deletion mutant ΔFgIlv2-4 and the complement strain ΔFgIlv2-1C was compared by streaking on CZ medium (using NaNO_3_ as the sole nitrogen source) containing single or multiple BCAAs at different concentrations. As shown in Fig. 2A, single addition of the three BCAAs Ile, Val and Leu could not restore the growth defects of ΔFgIlv2-4, and only multiple additions of Ile and Val at 5 mM could rescue the auxotrophic phenotype of ΔFgIlv2-4. Addition of 1.25, 2.5 mM Ile and Val in the medium could not rescue the growth defects of ΔFgIlv2-4, the concentration of minimal requirement of Ile and Val for normal growth should be higher than 5 mM each (Fig. 2B).

### Auxotrophic complementation of the *FgILV6* deletion mutant occurred only when arginine was the nitrogen source

Growth of the wild-type PH-1, the deletion mutant ΔFgIlv6-12 and the complement strain ΔFgIlv6-9C was also compared by streaking on CZ medium containing single or multiple BCAAs at different concentrations. However, addition of the 3 BCAAs could not restore the growth defects of ΔFgIlv6-12 even at high concentrations (20 mM) after 10 days incubation at 25 °C (data not shown).

**Figure 3:**
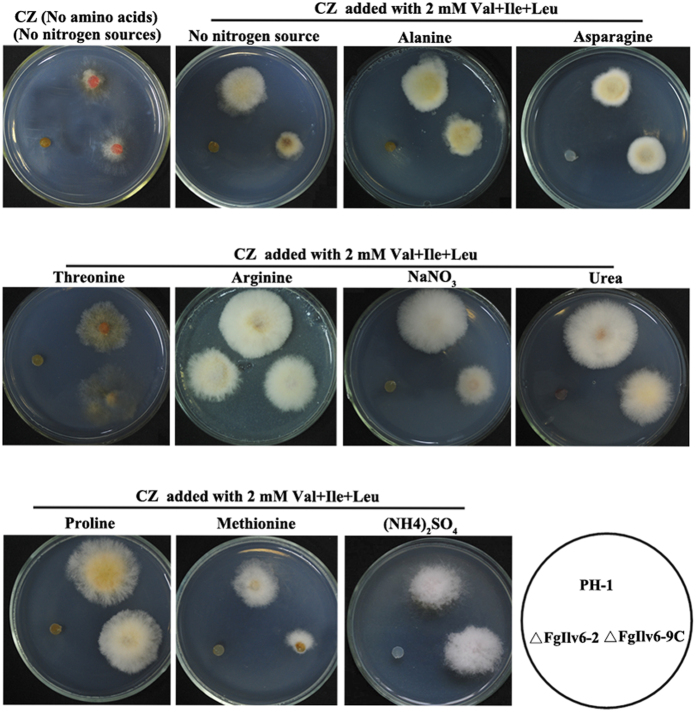
Growth of ΔFgIlv6-12 using different nitrogen sources. Mycelial morphology of PH-1, ΔFgIlv6-12 and ΔFgIlv6-9C cultured on CZ medium containing different nitrogen sources indicated in the figure and amended with Val, Ile and Leu at 2 mM after incubation at 25 °C for 2 days.

The inability of the *FgILV6* deletion mutant to grow on BCAA-containing medium could be attributed to the reduced BCAA permease activity being negatively regulated by nitrogen sources. Previous studies on the human pathogen *Cryptococcus neoformans* have revealed that the nitrogen regulation of amino acid transport was important for *ILV2* mutants to overcome BCAA auxotrophy^18^. Thus, we further checked the possibility of the nitrogen regulation of amino acid transport in *F. graminearum*. The wild-type PH-1, ΔFgIlv6-12 and ΔFgIlv6-9C were cultured on CZ medium containing ten different nitrogen sources added with three BCAAs at 2 mM. As shown in Fig. 3, the mycelial growth of the wild-type PH-1 and ΔFgIlv6-9C was very poor on CZ medium containing no nitrogen sources and no BCAAs, whereas addition of ten nitrogen sources restored poor mycelial growth to different levels. We were surprised to find that only when arginine (Arg) was used as a nitrogen source, accompanied by the simultaneous supplementation of three BCAAs at 2 mM each, was the auxotrophy of ΔFgIlv6-12 rescued (Fig. 3). Further analysis showed that exogenous addition of a single BCAA at 2 mM to the culture medium containing Arg as the nitrogen source could also rescue the auxotrophy of ΔFgIlv6-12 (data not shown).

**Figure 4:**
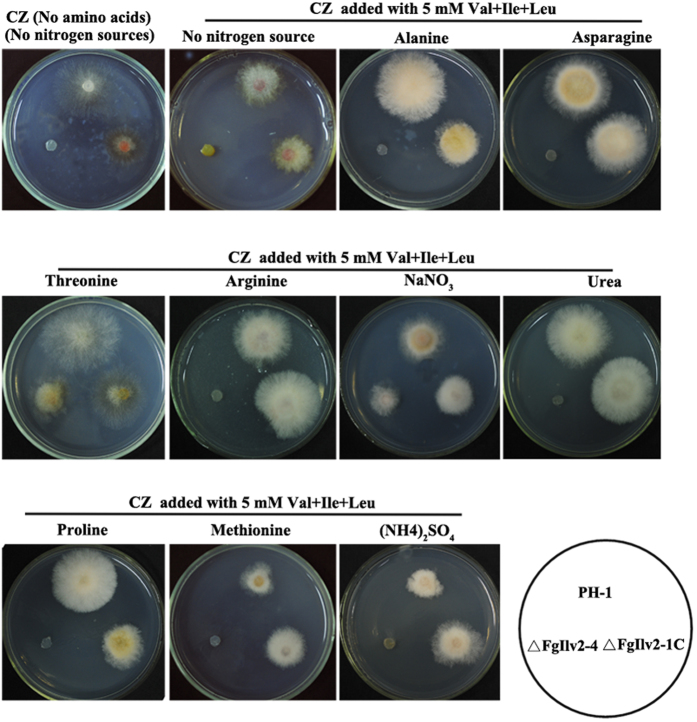
Growth of ΔFgIlv2-4 using different nitrogen sources. Mycelial morphology of PH-1, ΔFgIlv2-4 and ΔFgIlv2-1C cultured on CZ medium with different nitrogen sources indicated in the figure and amended with Val, Ile and Leu at 5 mM after incubation at 25 °C for 2 days.

Nitrogen sources of the culture medium also affected the growth of ΔFgIlv2-4 in the presence of exogenous Ile and Val. The wild-type PH-1, ΔFgIlv2-4 and ΔFgIlv2-1 C were cultured on CZ medium containing ten different nitrogen sources amended with Ile and Val at 5 mM, and results showed that only when threonine (Thr) or NaNO_3_ served as nitrogen source, would the exogenous addition of 5 mM Ile and Val meet the amino acid demands of ΔFgIlv2-4 (Fig. 4).

### *FgILV2* and *FgILV6* are involved in conidial morphogenesis

**Figure 5:**
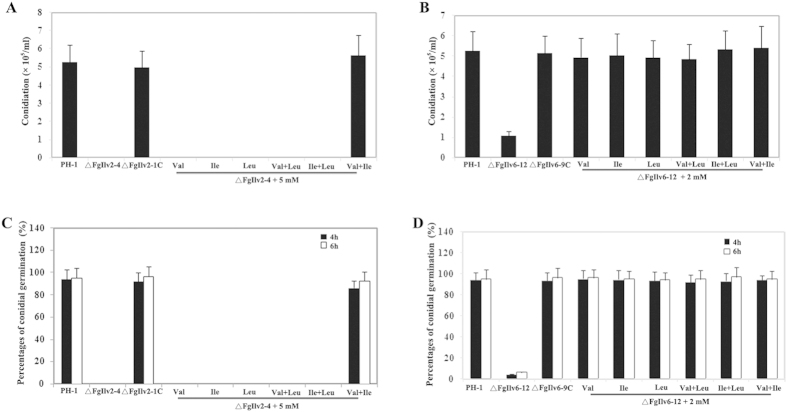
FgILV2 and FgILV6 are both required for conidial formation and germination in *F. graminearum*. (**A**) Comparison of conidiation among PH-1, ΔFgIlv2-4 and ΔFgIlv2-1C in MBL amended with or without amino acids. Line bars in each column denote standard errors of three repeated experiments. (**B**) Comparison of conidiation among PH-1, ΔFgIlv6-12 and ΔFgIlv6-9C in MBL amended with or without amino acids. Line bars in each column denote standard errors of three repeated experiments. (**C**) Conidia of PH-1, ΔFgIlv2-4 and ΔFgIlv2-1C harvested from MBL supplemented with 5 mM Val and Ile were re-suspended in 2% sucrose solution and cultured at 25 °C for 4 h and 6 h with or without addition of amino acids. Germination rate of each strain was calculated and line bars in each column denote standard errors of three repeated experiments. (**D**) Conidia of PH-1, ΔFgIlv6-12 and ΔFgIlv6-9C harvested from MBL were re-suspended in 2% sucrose solution and cultured at 25 °C for 4 h and 6 h with or without addition of amino acids. Germination rate of each strain was calculated and line bars in each column denote standard errors of three repeated experiments.

Disruption of both *FgILV2* and *FgILV6* affected conidial formation and germination. Conidial formation was completely blocked in ΔFgIlv2-4 when cultured in MBL (Fig. 5A). Genetic complementation of the full-length *FgILV2* or addition of 5 mM Ile and Val could rescue the conidial formation of ΔFgIlv2-4. For the conidial germination assays, considering ΔFgIlv2-4 could not produce conidia, a modified protocol was used. The addition of 5 mM Ile and Val to MBL allowed the production of conidia by ΔFgIlv2-4 to be used for germination assays. Like conidial formation, germination of the recovered conidia of ΔFgIlv2-4 was also completely blocked but could be overcome by supplementation of 5 mM Ile and Val to the germination buffer (Fig. 5C). ΔFgIlv6-12 produced significantly fewer conidia when compared to the wild-type PH-1 and ΔFgIlv6-9 C while conidial germination was also greatly reduced. Again, simultaneous addition of 2 mM optional BCAAs could rescue the conidial formation and germination defects of ΔFgIlv6-12 (Fig. 5B,D).

### *FgILV2* and *FgILV6* are involved in the mediation to various cellular stresses

**Figure 6:**
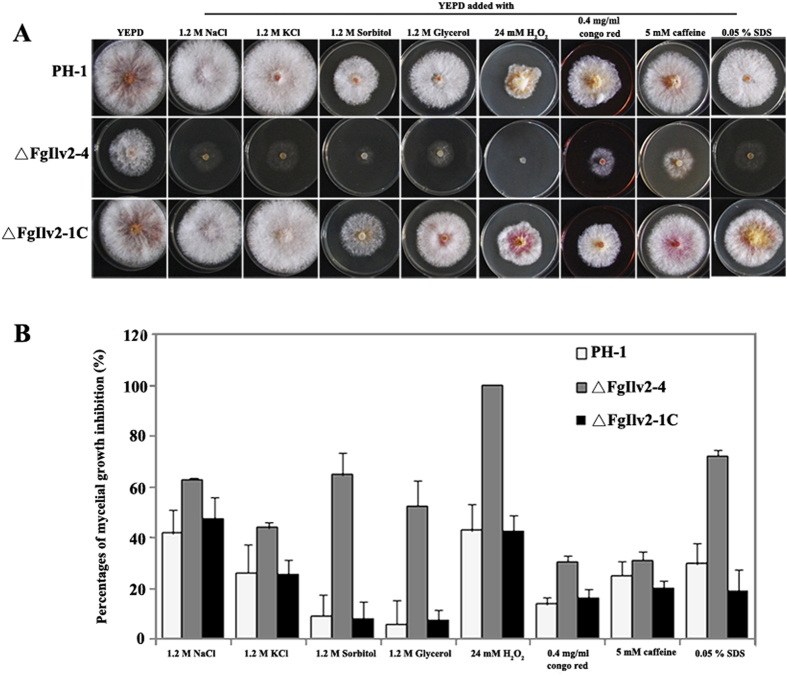
Morphology and mycelial inhibition of FgILV2 when subjected to various cellular stresses. (**A**) Colony morphology of PH-1, ΔFgIlv2-4 and ΔFgIlv2-1C cultured on various cellular stresses at concentrations described in the figure. (**B**) Comparisons of mycelial inhibition percentages of each strain grown on YEPD medium amended with various cellular stresses at concentrations described in the figure. Line bars in each column denote standard errors of three repeated experiments.

**Figure 7:**
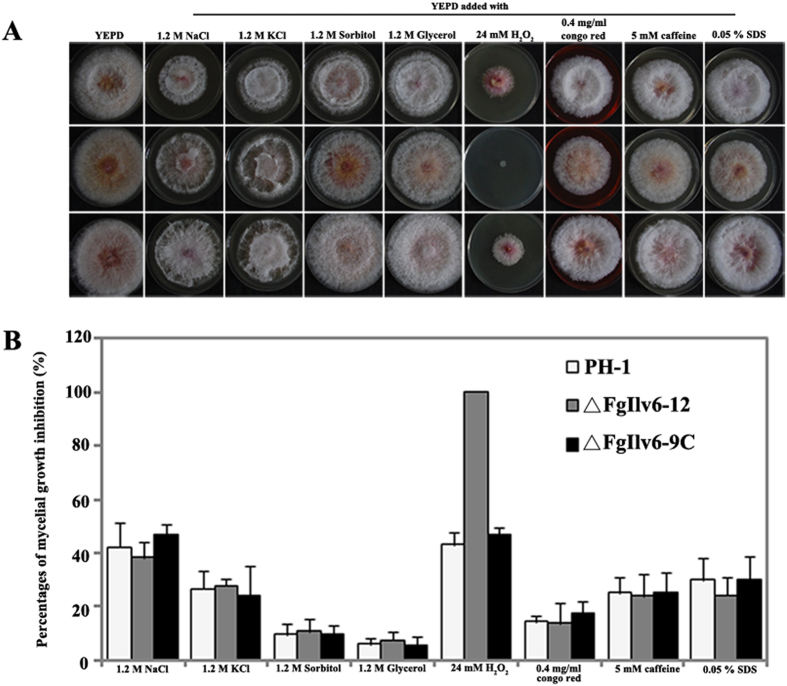
Morphology and mycelial inhibition of *FgILV6* when subjected to various cellular stresses. (**A**) Colony morphology of PH-1, ΔFgIlv6-12 and ΔFgIlv6-9C cultured on various cellular stresses at concentrations described in the figure. (**B**) Comparisons of mycelial inhibition percentages of each strain grown on YEPD medium amended with various cellular stresses at concentrations described in the figure. Line bars in each column denote standard errors of three repeated experiments.

Sensitivity assays to cellular stresses were conducted using YEPD medium as this medium provided all the nutrients needed by the deletion mutants to overcome their amino acid auxotrophy. As shown in Fig. 6A,B, deletion of *FgILV2* resulted in increased sensitivity to all eight cellular stresses including four osmotic stresses mediated by NaCl, KCl, D-sorbitol and glycerol at 1.2 M, the oxidative agent H_2_O_2_ at 12 mM, the cell wall inhibitors congo red at 0.4 mg/ml and caffeine at 5 mM, and the cell membrane disruptor 0.05% SDS. Sensitivity of ΔFgIlv6-12 did not show recognizable changes to the cellular stresses tested except for the oxidative agent H_2_O_2_, and, like ΔFgIlv2-4, ΔFgIlv6-12 could not survive when the culture medium was oxidative-stressed (Fig. 7A,B).

### *FgILV2* and *FgILV6* are required for full virulence and DON accumulation in *F. graminearum*

**Figure 8:**
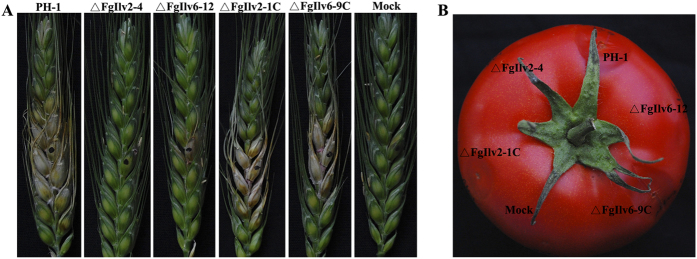
Both *FgILV2* and *FgILV6* are required for full virulence of *F. graminearum*. (**A**) Flowering wheat heads were point inoculated with a conidial suspension at 10^5^ conidia/ml of PH-1, ΔFgIlv2-4, ΔFgIlv2-1C, ΔFgIlv6-12 and ΔFgIlv6-9C. Infected wheat heads were photographed 10 days after inoculation. (**B**) Tomatoes were inoculated with a conidial suspension at 10^5^ conidia/ml of each strain and infected fruits were photographed 3 days after inoculation.

Results of the plant infection assays showed that disruption of both *FgILV2* and *FgILV6* resulted in significantly reduced virulence in flowering wheat heads and the non-host tomatoes. As shown in Fig. 8A,B, ΔFgIlv2-4 was non-pathogenic on both flowering wheat heads and tomatoes, whereas ΔFgIlv6-12 could infect the inoculated spikelet but lost aggressiveness on flowering wheat heads and could not cause water-soaked rot lesion on tomatoes.

**Figure 9:**
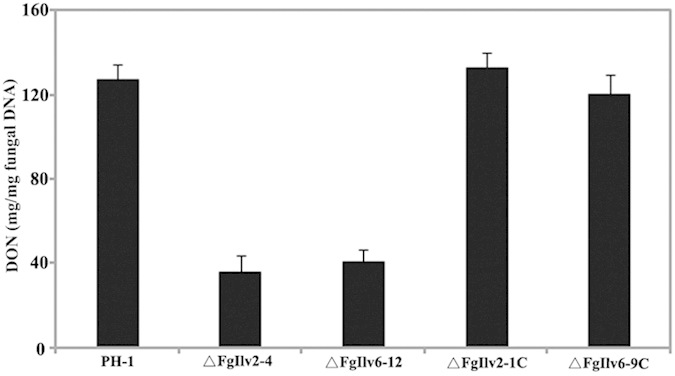
Deletion of *FgILV2* and *FgILV6* leads to reduced DON accumulation in wheat kernels in *F. graminearum*. The amounts of DON (mg/mg fungal DNA) produced by PH-1, ΔFgIlv2-4, ΔFgIlv2-1C, ΔFgIlv6-12 and ΔFgIlv6-9C in infected wheat kernels with bars denoting standard errors from three repeated experiments.

Previous studies have revealed the crucial roles of trichothecene production in the aggressiveness of *F. graminearum* during infection processes in wheat heads^24^,^25^. We checked if the disruption of *FgILV2* and *FgILV6* affects the production of the toxic trichothecene deoxynivalenol (DON), and as shown in Fig. 9, when cultured on sterilized wheat kernels for three weeks, the amounts of DON produced by the wild-type PH-1 were 3.6 and 3.2 fold higher than ΔFgIlv2-4 and ΔFgIlv6-12, respectively.

## Discussion

AHAS is the first shared enzyme in the BCAA biosynthetic pathway and is also known as the inhibitory target of the most widely used herbicides (AHAS inhibitors). Recent studies have revealed that certain AHAS-targeted herbicides exert inhibitory effects against several pathogenic bacteria and yeast^12^,^13^,^14^,^15^,^16^, indicating a promising potential of AHAS as an anti-microorganism target. Functional analyses in *S. cerevisiae, C. albicans, C. neoformans* and *M. oryzae* have suggested that the AHAS catalytic subunit-encoding gene *ILV2* has potential to be an antifungal target considering the phenotype defects caused by the disruption of *ILV2*^17^,^18^,^19^,^20^. In this study, disruption mutants of both the catalytic and regulatory subunits of AHAS in *F. graminearum* were BCAA-auxotrophic, indicating both AHAS subunits were essential components of BCAA biosynthesis in this fungus. The block in BCAA biosynthesis caused by the deletion of *FgILV2*/*6* resulted in defects in vegetative growth and conidiation and judging from the degree of severity of phenotypic defects displayed by the mutants, FgIlv2 may play a more important role in *F. graminearum* than FgIlv6. Additionally, reduced red pigmentation was observed in both deletion mutants and the down-regulation of the six red pigment biosynthetic genes^21^,^22^,^23^ (Table 1) may somehow explain this phenotypic defect.

In this study, we also found that BCAA absorption may be regulated by nitrogen sources under BCAA-starved conditions. Based on our results, either Thr, NaNO_3,_ or Arg are the preferred nitrogen sources for the function of BCAA permeases while other nitrogen sources tested may have a negative impact on BCAA permease activity. In a previous study in *C. neoformans*, the auxotrophy of an *ILV2* mutant was overcome by addition of Ile and Val only when proline, and not ammonium, was the nitrogen source^18^. In *M. oryzea*, addition of Ile and Val could meet the demands of the *MgILV2* deletion mutant and the auxotrophic defects of the *MgILV6* deletion mutant could be restored by only Val or by any two optional BCAAs when NaNO_3_ was the nitrogen source^20^. The role of BCAA permeases has been described in some Gram-positive bacteria since BCAAs not only are important nutrients for growth but also activate the function of CodY, a global transcriptional regulator^26^,^27^. Recently, Belitsky identified three BCAA permeases (BcaP, BraB and BrnQ) in *Bacillus subtilis* where all three are responsible for Ile, Val and Leu uptake and BcaP appears to be the most efficient BCAA permease^26^. In the human pathogen *Staphylococcus aureus*, three putative BCAA transporters (BrnQ1, BrnQ2 and BrnQ3) have been characterized and they displayed different substrate specificities; BrnQ1 is involved in uptake of all three BCAAs, BrnQ2 transports Ile, and BrnQ3 does not have a significant role in BCAA transport^27^.

The mechanism of BCAA acquisition, however, remains unexplored in filamentous fungi. In the genome database of *F. graminearum*, through homologous search, nine putative BCAA permease-encoding genes have been identified, and of these, three genes under accession number FGSG_10394.3, FGSG_02767.3 and FGSG_01675.3 showed the highest similarity and/or had an amino acid permease domain. Future work analyzing the possible roles of these BCAA permease-encoding genes in the wild type strain as well as in *ILV*-deleted background mutants will provide new insights in how different nitrogen sources affect BCAA uptake under BCAA-starved conditions.

Cellular stress assay results showed that both *FgILV2* and *FgILV6* deletion mutants showed increased sensitivity to the oxidative stress agent H_2_O_2_. Reduced tolerance to H_2_O_2_ was also found in our previously characterized *FgILV5* (encoding enzyme KARI, one step downstream of AHAS) mutant^28^, strengthening the hypothesis that certain components in BCAA biosynthesis are involved in the adaption to oxidative stresses. Besides the reduced tolerance to the oxidative stress by H_2_O_2_, disruption of *FgILV2*, but not *FgILV6*, caused increased sensitivity to other cellular stresses including osmotic stresses and cell wall and cell membrane inhibitors, indicating a more crucial role of FgIlv2 in the mediation of adaption to various cellular stresses.

Lost or reduced plant virulence could not be separated from the *in vitro* phenotypic defects of the mutants ΔFgIlv2-4 and ΔFgIlv6-12. Both mutants, when cultured on PDA medium or when infected into wheat heads, had lower growth when compared to the wild-type strain PH-1, indicating the limitation of acquiring amino acids by the fungus from the host during infection. It is also well accepted that reactive oxygen species play important roles in the host-pathogen interactions. Under plant pathogen attack, hosts employ an oxidative burst as early defense. The reduced tolerance of *FgILV2*/*6* mutants to H_2_O_2_ may partially account for the complete loss or reduced virulence in both flowering wheat heads and tomatoes. DON production may be another critical factor, as DON has been identified as a crucial virulence factor and plays an important role in the spread of the fungus during infection in wheat heads^24^,^25^. The significantly decreased DON production in *FgILV2*/*6* mutants could also be one of the reasons explaining the inability of spread within the spikelets during infection.

In summary, we have demonstrated that *FgILV2* and *FgILV6* encoding the catalytic and regulatory subunits of AHAS in *F. graminearum* are required for BCAA biosynthesis with their subsequent involvement in mycelial and conidial morphogenesis, adaption to cellular stresses, full virulence, and DON biosynthesis. These results are in agreement with our previously characterized BCAA biosynthetic gene deletion mutants, e.g., deletion mutants of *FgILV1* and *FgILV5* encoding the upstream and downstream enzymes of AHAS, respectively, which were BCAA- auxotrophic and displayed a series of phenotypic defects in *F. graminearum*^27^,^28^,^29^. These findings indicate the crucial role of the BCAA biosynthesic pathway in fungal growth and development in the filamentous fungi *F. graminearum* and imply that essential components in BCAA biosynthesis could make desirable targets for the development of novel fungicides.

## Materials and Methods

### Strains, culture media and growth conditions

*Fusarium graminearum* strain PH-1 was used as the parental wild type. To assess the mycelial growth and colony characteristics, the wild type strain and mutants were cultured on potato dextrose agar (PDA), yeast extract peptone glucose agar (YEPD), fructose gelatin agar (FGA) or czapek dox medium (CZ) plates supplemented with or without different amino acids and incubated at 25 °C. Colony diameter was measured every 12 h. Fungal biomass was measured by collecting mycelia from 2-day-old liquid culture, washing with sterilized water, drying at 65 °C in an oven, and weighing. *Escherichia coli* DH5α was cultured at 37 °C and used for normal bacterial transformations.

For the conidiation assay, six 5-mm mycelial plugs of each strain taken from the edge of a 3-day-old colony were inoculated in a 50-ml triangular flask containing 20 ml of mung bean liquid (MBL, 40 g mung beans boiled in 1 L water for 20 min, and then filtered through cheesecloth) supplemented with different amino acids for conidiation. The flasks were incubated at 25 °C and 180 rpm for 4 days in a shaker^30^. Haemocytometer counts were made to determine the amount of conidia in the broth for each sample. Additionally, conidial germination was carried out by re-suspending conidia in 2% sucrose solutions amended with or without amino acids at 25 °C for 4 h and 6 h and examining under a Nikon ECLIPSE E100 microscope (Nikon Co., Tokyo, Japan). Each experiment was carried out with three replicates and performed three times.

### Sequencing of *FgILV2* and *FgILV6* in *F. graminearum*

With BLASTP search at the *Fusarium* Comparative Database site (available at http://www.broad.mit.edu/annotation/genome/fusarium_graminearum/Home.html) using the amino acid sequence of *S. cerevisiae* AHAS catalytic subunit Ilv2 (*Saccharomyces* database accession number YMR108W) and regulatory subunit Ilv6 (YCL009C) as query, we identified *F. graminearum* AHAS catalytic subunit homolog *FgILV2*, Broad Institute *Fusarium* comparative database accession number FGSG_01086.3, and regulatory subunit homolog *FgILV6*, FGSG_06282.3.

To validate the existence of introns in both genes, RNA was extracted from mycelia of wild type PH-1 using TaKaRa RNAiso Reagent (TaKaRa Biotech. Co., Dalian, China), and used for reverse transcription with PrimeScript^TM^ RT reagent kit (TaKaRa Biotech. Co., Dalian, China) and the primer oligo(dT)_18_. PCR amplification with primer pair Ilv2-all-F + Ilv2-all-R and Ilv6-all-F + Ilv6-all-R (Table S1) were performed for *FgILV2* and *FgILV6* respectively using cDNA and genomic DNA as templates. PCR amplifications were conducted using the following parameters: initial denaturation at 95 °C for 3 min, followed by 30 cycles of denaturation at 94 °C for 45 s, annealing at 53 °C for 45 s, extension at 72 °C for 1.5 min, and final extension at 72 °C for 10 min. PCR products were separated on 1% agarose gels in Tris-acetate (TAE) buffer and photographed after staining with ethidium bromide. The resultant PCR product was purified, cloned and sequenced.

### Targeted disruption of *FgILV2* and *FgILV6*

To obtain *FgILV2* deletion mutants, plasmid pBS-ilv2-Del was constructed. Two flanking sequences of *FgILV2* were amplified from the genomic DNA of the wild-type PH-1 and inserted into the left and right sides of a hygromycin resistance gene (*HPH*) in the multiple cloning site of the plasmid pBS-HPH1 (Supplementary Figure 2A)^31^. Using primer pair A1 + A2 (Table S1), a 991-bp upstream flanking sequence fragment of *FgILV2* was amplified from PH-1 genomic DNA and inserted into *Kpn* I-*Xho* I sites of the pBS-HPH1 vector to generate plasmid pBS-ilv2-up. Subsequently, a 992-bp downstream flanking sequence fragment of *FgILV2* amplified from PH-1 genomic DNA using the primers A3 + A4 (Table S1) was inserted into *Bam*H I-*Sac* I sites of the pBS-ilv2-up vector to generate plasmid pBS-ilv2-Del. Similar to pBS-ilv2-Del, we constructed the *FgILV6* deletion vector pBS-ilv6-Del (see Supplementary Figure 3A for details). Finally, the 3486-bp fragment containing the ilv2-upstream-HPH-ilv2-downstream cassette or the 3642-bp fragment containing ilv6-upstream-HPH-ilv6-downstream cassette was obtained by PCR amplification with primer pair A1 + A4 or B1 + B4 using diluted plasmid pBS-ilv2-Del or pBS-ilv6-Del as templates, respectively. PCR products were separated on 1% agarose gels in TAE buffer and photographed after staining with ethidium bromide. The resultant PCR product was purified and stored at −20 °C for protoplast transformation.

The PEG-mediated protoplast transformation of *F. graminearum* was performed as described previously^32^. For selective growth of transformants, PDA medium supplemented with hygromycin (100 mg/L) was used. After single spore isolation, transformants ΔFgIlv2-4 and ΔFgIlv6-12 were selected for further analysis and were kept at 4 °C for further following experiments.

### Complementation of *FgILV2* and *FgILV6* deletion mutants

To confirm that the phenotype of *FgILV2* or *FgILV6* deletion mutants is due to disruption of the gene, genetic complementation was performed. A 3626-bp of full length *FgILV2,* including the 579-bp upstream and 530-bp terminator regions, was amplified using primer pair ilv2-com-F + ilv2-com-R (Table S1) from genomic DNA of wild type strain PH-1. This fragment was cloned into the *Bam*H I-*Pst* I sites of pCAMBIA1300-NEO to generate the complement plasmid pCA-ilv2-Com (Supplementary Figure 2A). Similar to pCA-ilv2-Com, we constructed the *FgILV6* complementary vector pCA-ilv6-Com (Supplementary Figure 3A). A 2987-bp of full length *FgILV6* including a 1135-bp upstream and 863-bp terminator region was amplified using primer pair ilv6-com-F + ilv6-com-R (Table S1) from genomic DNA of wild type strain PH-1, and the subsequent fragment was cloned into the *Bam*H I - *Pst* I sites of pCAMBIA1300-NEO to generate the complement plasmid pCA-ilv6-Com.

Transformation of ΔFgIlv2-4 or ΔFgIlv6-12 with the full-length *FgILV2* gene or *FgILV6* gene was conducted as described above except that G418 sulfate (100 mg/L) was used as a selection agent. Transformants ΔFgIlv2-1C and ΔFgIlv6-9C were selected for further analysis and were kept at 4 °C for further following experiments.

### Amino acid assays

Exogenous amino acids were supplemented to solid or liquid media at different concentrations indicated in figures to further elucidate the effects of leucine, isoleucine and valine auxotrophy on mycelial growth, conidial formation and germination. Experiments were performed in triplicate.

### Plant infection assays

Conidia were harvested by centrifugation of the 4-day-old liquid culture filtered through three layer autoclaved gauze of the wild type and mutants and were resuspended in 0.01% (vol/vol) Tween 20 with or without amino acids and adjusted to 10^5^ conidia/ml. A pathogenicity test was performed with single floret injection method as previously described^33^. Briefly, 10 μl of conidial spores (10^5^/ml) were injected into a single floret in the central section spikelet of single flowering wheat heads of susceptible cultivar Jimai 19 at early anthesis, and 10 spikes were used for each strain. A control inoculation with 10 μl only Tween20 was conducted. Infected spikelets in each inoculated wheat head were recorded 10 days after inoculation. The experiment was repeated three times. To examine the ability to colonize tomato, a 10 μl aliquot of conidial suspension was injected into the wounded tomato after surface sterilization. There were five replicates for each strain. Inoculated tomatoes were incubated at 25 °C and 100% humidity with 12 h of daylight, and were photographed 3 days after inoculation. The experiment was repeated three times.

### Determination of sensitivity of the *FgILV2* and *FgILV6* mutants to cell stresses

Mycelial growth tests were performed on YEPD plates supplemented with the following chemicals: osmotic stress (NaCl, D-sorbitol, KCl and glycerol); oxidative stress (H_2_O_2_); cell wall inhibitors (congo red and caffeine); cell membrane disruptor (SDS) at the concentrations indicated in the figures. The percentage of inhibition of mycelial radial growth (PIMG) was calculated using the formula PIMG = [(C-N)/(C-5)] * 100, where C is the colony diameter of the non-treatment, and N is that of the chemical treatment. Each experiment was carried out with three replicates and the experiments were repeated twice.

### Determination of DON production

Diseased wheat kernels were harvested from inoculated spikelets 20 days after inoculation and used for the assays of DON production using a previously described protocol^34^,^35^. The amount of DON in each sample was determined using a high-performance liquid chromatography-mass spectrometer/mass spectrometer (HPLC-MS/MS) system (Agilent1100-6410, Agilent Technologies, Palo Alto, Calif.). Briefly, ten grams of wheat powder sample were extracted with 40 mL of acetonitrile:water:acetic acid (79:20:1 v/v/v) at 180 rpm for 30 min. After centrifugation at 3000 rpm for 10 min, 0.5 mL of each final extract was diluted with acetonitrile:water:acetic acid (20:79:1 v/v/v) and filtered through a nylon filter (13 mm in diameter, 0.22-lm pore size). The LC-MS/MS system consisted of an Agilent 1200 HPLC, an Agilent 6410B triple-quadrupole mass spectrometer, and an Agilent MassHunter Workstation running Qualitative Analysis Version B.01.03 software for data acquisition and analysis. The analytical column used was an XDB-C18 (2.1 × 150 mm, 3.5-μm bead diameter; Agilent), and the column temperature was held at 30 °C. Nitrogen was used as drying gas (10 L/min). The capillary voltage was 4 kV, the nebulizer pressure 25 psi, and the drying gas temperature 350 °C. DON was analyzed via multiple reactions monitoring (MRM). Mass spectrometric parameters of DON and the composition of the mobile phase were according to a previously described detection method^36^.

Additionally, the amount of *F. graminearum* DNA in each sample was determined using a quantitative realtime PCR method^37^. Briefly, ground wheat sample (50 mg each) was transferred into a 1.5 ml microtube and further mixed with a motor-driven glass pestle in 1 ml of the lysis buffer (200 mmol l^−1^ Tris-HCl, 50 mmol l^−1^ ethylenediaminetetraacetic acid, 200 mmol l^−1^ NaCl, 1% (w/v) n-lauroylsarcosine sodium salt and 2% (w/v) polyvinyl polypyrrolidone, pH 8.0). The mixture was incubated for 10 min at room temperature before being centrifuged at 18,000 g for 5 min at 4 °C. The supernatant (800 μl) was transferred to a new microtube and mixed with an equal volume of isopropanol. Then the DNA pellet was precipitated by centrifugation at 18,000 g for 2 min at 4 °C. After a wash with 70% ethanol, the DNA pellet was air-dried and dissolved in 100 μl of 100 mmol l^−1^ Tris-HCl buffer (pH 8.0). To remove PCR inhibitors released from wheat powder, the DNA extract was further purified using an UNIQ gel extraction kit (Sangon Co., Shanghai, China). The final DNA was suspended in 20 μl elution buffer, and a 2 μl aliquot of DNA was used for each real-time PCR amplification. To create a standard curve, serial dilutions of *F. graminearum* DNA (ranging from 0.5 pg to 50 ng) spiked with 2 ng wheat DNA extracted from healthy wheat were used as template for real-time PCR with primer pair FgF + FgR. The real-time PCR amplifications were conducted as described above. Three replicates were used for each sample, and the experiment was performed three times.

### Analysis of gene expression level

Total RNA of the parental strain PH-1 and deletion mutants were extracted and cDNA was reverse transcribed using a protocol as described above. Expression of the pigment genes was determined by quantitative real-time PCR (RT-PCR) with primer pair Pks12-RT-F + Pks12-RT-R, Gip1-RT-F + Gip1-RT-R, Gip2-RT-F + Gip2-RT-R, AurF-RT-F + AurF-RT-R, AurR_2_-RT-F + AurR_2_-RT-R, AurO-RT-F + AurO-RT-R, respectively (Table S1). The RT-PCR amplifications were performed in a Rotor Gene^TM^ 6000 (Corbett Research, Australia) using the SYBR Green I fluorescent dye detection. Amplifications were conducted in a 20 μl volume containing 10 μl of iQ SYBR Green Supermix (Bio-Rad Laboratories, Hercules, California, USA), 1 μl of reverse transcription product and 0.5 μl of each primer (Table S1). There were three replicates for each sample. The real-time PCR amplifications were performed with the following parameters: an initial preheat at 95 °C for 2 min, followed by 35 cycles at 95 °C for 15 s, 60 °C for 20 s, 72 °C for 20 s, and 75 °C for 3 s in order to quantify the fluorescence at a temperature above the denaturation of primer-dimers. Once amplifications were completed, melting curves were obtained to identify PCR products. For each sample, PCR amplifications with primer pair Fgactin-F + Fgactin-R (Table S1) for the quantification of expression of actin gene were performed as a reference. The experiment was repeated three times. The relative expression levels of genes in the wild-type strain and deletion mutants were calculated using the 2^-ΔΔCt^ method^38^.

### Standard molecular methods

Fungal genomic DNA was extracted using a previously published protocol^39^. Plasmid DNA was isolated using a plasmid miniprep purification kit (BioDev Co., Beijing, China). Southern blot hybridization analysis of *FgILV2* and *FgILV6* gene in each strain of *F. graminearum* was performed using probes as indicated in Figure S1 and Figure S2. Each probe was labeled with digoxigenin (DIG) using a high prime DNA labeling and detection starter kit II according to according to the manufacturer’s instructions (Roche Diagnostics; Mannheim, Germany).

## Additional Information

**How to cite this article**: Liu, X. *et al.* Acetohydroxyacid synthase FgIlv2 and FgIlv6 are involved in BCAA biosynthesis, mycelial and conidial morphogenesis and full virulence in *Fusarium graminearum. Sci. Rep.* **5**, 16315; doi: 10.1038/srep16315 (2015).

## Supplementary Material

Supplementary Information

## Figures and Tables

**Table 1:** Expression changes of the genes involved in red pigment biosynthesis in *F. graminearum FgILV2* deletion mutant ΔFgIlv2-4 and *FgILV6* deletion mutant ΔFgIlv6-12 as detected by quantitative real time-PCR.

Gene (Broad accession number)	Putative function	Average change fold in ΔFgIlv2-4* (mean ± SD)	Average change fold in ΔFgIlv6-12* (mean ± SD)
*PKS12* FGSG_02324	hypothetical protein similar to type I polyketide synthase	0.295 ± 0.02	0.257 ± 0.02
*Gip1* FGSG_02328	hypothetical protein similar to brown 2 (dimerize two 9-hydroxyrubrofusarin)	0.325 ± 0.01	0.140 ± 0.02
*Gip2* FGSG_02320	hypothetical protein similar to C6 zinc finger protein (Positive acting transcription factor for the aurofusarin gene cluster)	0.262 ± 0.03	0.504 ± 0.09
*AurJ* FGSG_02326	conserved hypothetical protein (O-methyltransferase: converts nor-rubrofusarin into rubrofusarin)	0.666 ± 0.04	0.200 ± 0.02
*AurF* FGSG_02327	conserved hypothetical protein (Monooxygenase: converts rubrofusarin into 9-hydroxyrubrofusarin)	0.571 ± 0.05	0.186 ± 0.04
*AurR2* FGSG_02323	hypothetical protein similar to AurR2	0.415 ± 0.06	0.279 ± 0.01

^*^The relative expression of each red pigment biosynthetic gene in the deletion mutant ΔFgIlv2-4 or ΔFgIlv6-12 was relative to amount of cDNA of each corresponding gene in the wild type progenitor PH-1. Data are means ± SD. There were significant differences between the relative expression (fold changes) of each red pigment biosynthetic among the wild type PH-1 and ΔFgIlv2-4 or ΔFgIlv6-12 (p < 0.05).

## References

[b1] Goswami R. S. & Kistler H. C. Heading for disaster: *Fusarium graminearum* on cereal crops. Mol.Plant Pathol. 5, 515–525 (2004). 20565626 10.1111/j.1364-3703.2004.00252.x

[b2] Wang J. H., Ndoye M., Zhang J. B., Li H. P. & Liao Y. C. Population structure and genetic diversity of the *Fusarium graminearum* species complex. Toxins 3, 1020–1037 (2011). 22069755 10.3390/toxins3081020 PMC3202863

[b3] Desjardins A. E. & Proctor R. H. Molecular biology of *Fusarium* mycotoxins. Int. J.Food Microbiol. 119, 47–50 (2007). 17707105 10.1016/j.ijfoodmicro.2007.07.024

[b4] Windels C. E. Economic and social impacts of fusarium head blight: changing farms and rural communities in the northern great plains. Phytopathology 90, 17–21 (2000). 18944567 10.1094/PHYTO.2000.90.1.17

[b5] Qu B. *et al.* Geographic distribution and genetic diversity of *Fusarium graminearum* and *F. asiaticum* on wheat spikes throughout China. Plant Pathol. 57, 15–24 (2008).

[b6] Liu X., Yin Y. N., Wu J. B., Jiang J. H. & Ma Z. H. Identification and characterization of carbendazim-resistant isolates of *Gibberella zeae*. Plant Dis. 94, 1137–1142 (2010). 30743730 10.1094/PDIS-94-9-1137

[b7] Shaner D. L., Anderson P. C. & Stidham M. A. Imidazolinones potent inhibitors of acetohydroxyacid synthase. Plant Physiol. 76, 545–546 (1984). 16663878 10.1104/pp.76.2.545 PMC1064324

[b8] Kohlhaw G. B. Leucine biosynthesis in fungi: entering metabolism through the back door. Microbiol.Molecul Biol. R. 67, 1–15 (2003). 10.1128/MMBR.67.1.1-15.2003 PMC150519 12626680

[b9] Tan S., Evans R. & Singh B. Herbicidal inhibitors of amino acid biosynthesis and herbicide-tolerant crops. Amino acids 30, 195–204 (2006). 16547651 10.1007/s00726-005-0254-1

[b10] Duggleby R. G., McCourt J. A. & Guddat L. W. Structure and mechanism of inhibition of plant acetohydroxyacid synthase. Plant Physiol.Biochem. 46, 309–324 (2008). 18234503 10.1016/j.plaphy.2007.12.004

[b11] Binder S. Branched-chain amino acid metabolism in *Arabidopsis thaliana*. The Arabidopsis book 8, e0137 (2010). 22303262 10.1199/tab.0137 PMC3244963

[b12] LaRossa R. A. & Schloss J. V. The sulfonylurea herbicide sulfometuron methyl is an extremely potent and selective inhibitor of acetolactate synthase in *Salmonella typhimurium*. J.Biol Chem 259, 8753–8757 (1984). 6378902

[b13] Grandoni J. A., Marta P. T. & Schloss J. V. Inhibitors of branched-chain amino acid biosynthesis as potential antituberculosis agents. J. Antimicrob.Chemoth. 42, 475–482 (1998). 10.1093/jac/42.4.475 9818746

[b14] Zohar Y., Einav M., Chipman D. M. & Barak Z. Acetohydroxyacid synthase from *Mycobacterium avium* and its inhibition by sulfonylureas and imidazolinones. Biochimica et biophysica acta 1649, 97–105 (2003). 12818195 10.1016/s1570-9639(03)00160-2

[b15] Kreisberg J. F. *et al.* Growth inhibition of pathogenic bacteria by sulfonylurea herbicides. Antimicrob.Agents Chemother. 57, 1513–1517 (2013). 23263008 10.1128/AAC.02327-12 PMC3591922

[b16] Lee Y. T. *et al.* Sulfonylureas have antifungal activity and are potent inhibitors of *Candida albicans* acetohydroxyacid synthase. J. Med. Chem. 56, 210–219 (2013). 23237384 10.1021/jm301501k

[b17] Kingsbury J. M., Goldstein A. L. & McCusker J. H. Role of nitrogen and carbon transport, regulation, and metabolism genes for *Saccharomyces cerevisiae* survival *in vivo*. Eukaryot. cell 5, 816–824 (2006). 16682459 10.1128/EC.5.5.816-824.2006 PMC1459679

[b18] Kingsbury J. M., Yang Z., Ganous T. M., Cox G. M. & McCusker J. H. *Cryptococcus neoformans* Ilv2p confers resistance to sulfometuron methyl and is required for survival at 37 degrees C and *in vivo*. Microbiology 150, 1547–1558 (2004). 15133116 10.1099/mic.0.26928-0

[b19] Kingsbury J. M. & McCusker J. H. Cytocidal amino acid starvation of *Saccharomyces cerevisiae* and *Candida albicans* acetolactate synthase (ilv2Δ) mutants is influenced by the carbon source and rapamycin. Microbiology 156, 929–939 (2010). 20019084 10.1099/mic.0.034348-0 PMC2841795

[b20] Du Y. *et al.* Acetolactate synthases MoIlv2 and MoIlv6 are required for infection-related morphogenesis in Magnaporthe oryzae. Mol. Plant Pathol. 14, 870–884 (2013). 23782532 10.1111/mpp.12053 PMC6638861

[b21] Kim J. E. *et al.* Putative polyketide synthase and laccase genes for biosynthesis of aurofusarin in *Gibberella zeae*. Appl.Environ.Microbiol. 71, 1701–1708 (2005). 15811992 10.1128/AEM.71.4.1701-1708.2005 PMC1082506

[b22] Kim J. E. *et al.* GIP2, a putative transcription factor that regulates the aurofusarin biosynthetic gene cluster in *Gibberella zeae*. Appl.Environ.Microbiol. 72, 1645–1652 (2006). 16461721 10.1128/AEM.72.2.1645-1652.2006 PMC1392934

[b23] Malz S. *et al.* Identification of a gene cluster responsible for the biosynthesis of aurofusarin in the *Fusarium graminearum* species complex. Fungal Genet. Biol. 42, 420–433 (2005). 15809006 10.1016/j.fgb.2005.01.010

[b24] Jansen C. *et al.* Infection patterns in barley and wheat spikes inoculated with wild-type and trichodiene synthase gene disrupted *Fusarium graminearum*. Pro. Nati. Acad. Sci. USA 102, 16892–16897 (2005). 10.1073/pnas.0508467102 PMC1283850 16263921

[b25] Proctor R. H., Hohn T. M. & McCormick S. P. Reduced virulence of *Gibberella zeae* caused by disruption of a trichothecene toxin biosynthetic gene. Mol. Plant Microbe Interact. 8, 593–601 (1995). 8589414 10.1094/mpmi-8-0593

[b26] Belitsky B. R. Role of branched-chain amino acid transport in *Bacillus subtilis* CodY activity. J. Bacteriol. 197, 1330–1338 (2015). 25645558 10.1128/JB.02563-14 PMC4372739

[b27] Kaiser J. C., Omer S., Sheldon J. R., Welch I. & Heinrichs D. E. Role of BrnQ1 and BrnQ2 in branched-chain amino acid transport and virulence in *Staphylococcus aureus*. Infect Immun. 83, 1019–1029 (2015). 25547798 10.1128/IAI.02542-14 PMC4333469

[b28] Liu X., Wang J., Xu J. H. & Shi J. R. FgIlv5 is required for branched-chain amino acid biosynthesis and full virulence in *Fusarium graminearum*. Microbiology 160, 692–702 (2014). 24493249 10.1099/mic.0.075333-0

[b29] Liu X. *et al.* Involvement of threonine deaminase FgIlv1 in isoleucine biosynthesis and full virulence in *Fusarium graminearum*. Curr. Genet. 10.1007/s00294-014-0444-z (2014). 25129826

[b30] Bai G. H., Desjardins A. E. & Plattner R. D. Deoxynivalenol-nonproducing *Fusarium graminearum* causes initial infection, but does not cause disease spread in wheat spikes. Mycopathologia 153, 91–98 (2002). 12000132 10.1023/a:1014419323550

[b31] Liu X., Fu J., Yun Y. Z., Yin Y. N. & Ma Z. H. A sterol C-14 reductase encoded by *FgERG24B* is responsible for the intrinsic resistance of *Fusarium graminearum* to amine fungicides. Microbiology 157, 1665–1675 (2011). 21436218 10.1099/mic.0.045690-0

[b32] Liu Z. & Friesen T. L. Polyethylene glycol (PEG)-mediated transformation in filamentous fungal pathogens. Methods Mol. Biol. 835, 365–375 (2012). 22183664 10.1007/978-1-61779-501-5_21

[b33] Wu A. B., Li H. P., Zhao C. S. & Liao Y. C. Comparative pathogenicity of *Fusarium graminearum* isolates from China revealed by wheat coleoptile and floret inoculations. Mycopathologia 160, 75–83 (2005). 16160772 10.1007/s11046-005-1153-4

[b34] Bluhm B. H., Zhao X., Flaherty J. E., Xu J. R. & Dunkle L. D. RAS2 regulates growth and pathogenesis in Fusarium graminearum. Mol. Plant Microbe Interact. 20, 627–636 (2007). 17555271 10.1094/MPMI-20-6-0627

[b35] Sasanya J. J., Hall C. & Wolf-Hall C. Analysis of deoxynivalenol, masked deoxynivalenol, and *Fusarium graminearum* pigment in wheat samples, using liquid chromatography-UV-mass spectrometry. J. Food Prot. 71, 1205–1213 (2008). 18592747 10.4315/0362-028x-71.6.1205

[b36] Soleimany G. *et al.* Bone mineral changes and cardiovascular effects among female athletes with chronic menstrual dysfunction. Asian J. Sports Med. 3, 53–58 (2012). 22461966 10.5812/asjsm.34730 PMC3307967

[b37] Yin Y. N., Liu X. & Ma Z. H. Simultaneous detection of *Fusarium asiaticum* and *Fusarium graminearum* in wheat seeds using a real-time PCR method. Lett. Appl. Microbiol. 48, 680–686 (2009). 19413810 10.1111/j.1472-765X.2009.02595.x

[b38] Livak K. J. & Schmittgen T. D. Analysis of relative gene expression data using real-time quantitative PCR and the 2(-^ΔΔ^C(T)) Method. Methods 25, 402–408 (2001). 11846609 10.1006/meth.2001.1262

[b39] Zhan J., Mundt C. C. & McDonald B. A. Using restriction fragment length polymorphisms to assess temporal variation and estimate the number of ascospores that initiate epidemics in field populations of *Mycosphaerella graminicola*. Phytopathology 91, 1011–1017 (2001). 18944129 10.1094/PHYTO.2001.91.10.1011

