# Supplementary material for: Acetohydroxyacid synthase FgIlv2 and FgIlv6 are involved in BCAA biosynthesis, mycelial and conidial morphogenesis, and full virulence in Fusarium graminearum
Source: Sci Rep. 2015 Nov 10;5:16315. doi: 10.1038/srep16315 (PMC4639788; doi:10.1038/srep16315)
Supplement: Supplementary Information [file srep16315-s1.pdf]

Acetohydroxyacid synthase FgIlv2 and FgIlv6 are involved in BCAA biosynthesis, mycelial and conidial morphogenesis and full virulence in *Fusarium graminearum*

Xin Liu[1,2], Qi Han[1], Jianhong Xu[1], Jian Wang[1] & Jianrong Shi[1,2]

[1]State Key Laboratory Breeding Base of Food Quality and Safety in Jiangsu Province; Key Laboratory of Control Technology and Standard for Agro-product Safety and Quality, Ministry of Agriculture; Key Laboratory of Agro-product Safety Risk Evaluation (Nanjing), Ministry of Agriculture; Institute of Food Quality and Safety, Jiangsu Academy of Agricultural Sciences, 210014, China, [2]Collaborative Innovation Center for Modern Grain Circulation and Safety.

Correspondence: Jianrong Shi, shiji@jaas.ac.cn

Supplementary figure captions

Figure S1. Phylogenetic tree generated by using the neighbor-joining method with Mega 4.1 software on the basis of deduced amino acid sequences of FgIlv2 from *Fusarium graminearum* strain PH-1 (indicated in the blank boxes), and those from *Aspergillus fumigatus* (GenBank accession no. EDP52715.1), *A. nidulans* (CBF76400.1), *Arabidopsis thaliana* (NP_190425.1), *Candida albicans* (XP_721692.1), *F. fujikuroi* (CCT73666.1), *F. oxysporum* (EGU74990.1), *F. verticillioides* (EWG51198.1), *Magnaporthe oryzae* (AHJ61470.1), *Penicillium digitatum* (EKV05884.1), *Saccharomyces cerevisiae* (NP_013826.1), and *Sclerotinia sclerotiorum* (XP_001592310.1)(left); FgIlv6 from *F. graminearum* strain PH-1 and those from *A. fumigatus* (XP_752105.1), *A. nidulans* (XP_662034.1), *A. thaliana* (NP_850173.2), *C. albicans* (EEQ45326.1), *F. fujikuroi* (CCT67313.1), *F. oxysporum* (EGU76155.1), *F. verticillioides* (EWG13519.1), *M. oryzae* (XP_003717830.1), *P. digitatum* (EKV19701.1), *S.cerevisiae* (NP_009918.1), and *S. sclerotiorum* (XP_001595836.1)(right).

Figure S2. Targeted disruption and complementation of *FgILV2*. **(A)** Gene replacement and complement strategy for *FgILV2* gene. Primer binding sites are indicated by arrows (see Table S1 for the primer sequences). **(B)** Reverse transcription PCR analysis of *FgILV2* expression in PH-1, ΔFgIlv2-4 and ΔFgIlv2-1C using cDNA as template. NCK is a negative control without template cDNA in the PCR amplification. (**C**) Southern blot hybridization analysis of PH-1, ΔFgIlv2-4 and ΔFgIlv2-1C using a 992-bp *FgILV2* downstream fragment as a probe. Genomic DNA preparation of each strain was digested with *Eco*R I.

Figure S2. Targeted disruption and complementation of *FgILV6*. **(A)** Gene replacement and complement strategy for *FgILV6* gene. Primer binding sites are indicated by arrows (see Table S1 for the primer sequences). **(B)** Reverse transcription PCR analysis of *FgILV6* expression in PH-1, ΔFgIlv6-12 and ΔFgIlv6-9C using cDNA as template. NCK is a negative control without template cDNA in the PCR amplification. (**C**) Southern blot hybridization analysis of PH-1, ΔFgIlv6-12 and ΔFgIlv6-9C using a 989-bp *FgILV6*

upstream fragment as a probe. Genomic DNA preparation of each strain was digested with *Aat* Ⅱ

Figure S1

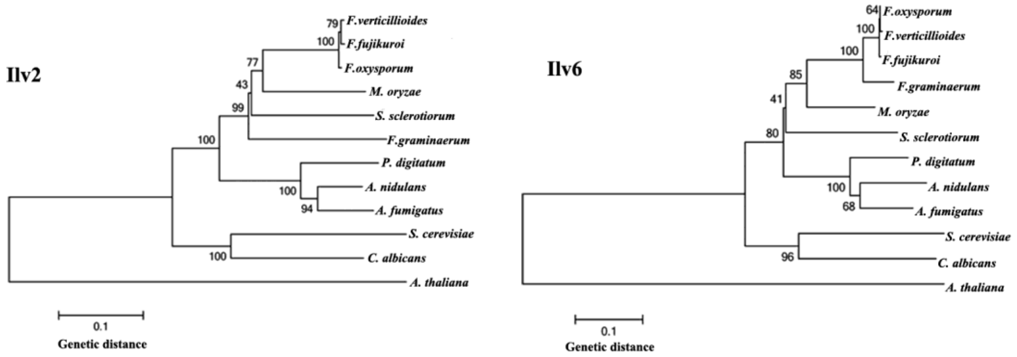

Figure S2

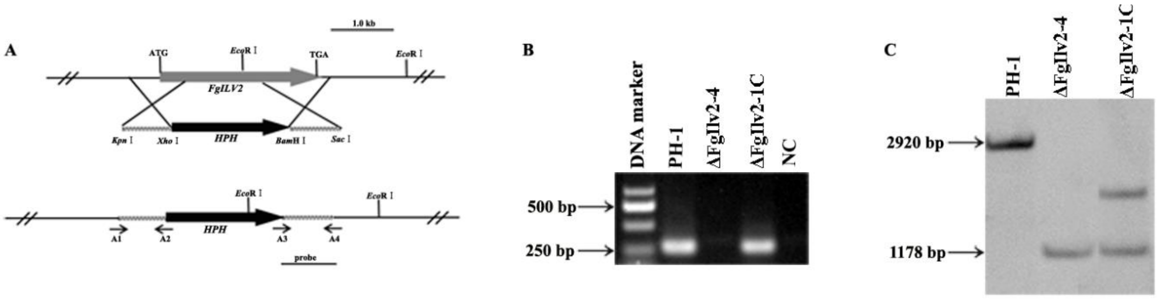

Figure S3

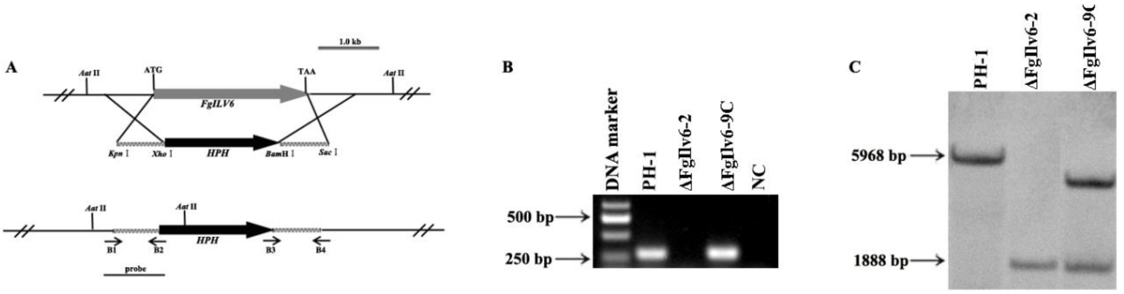

Table S1. Oligonucleotide primers used in this study and their relevant characteristics.

Primer	Sequence(5'-3')	Relevant characteristics
A1<br>A2	ATggtaccGGTTTATGGCATTTTGGTCAG<br>ATctcgagCACCGGACTTTCCAATGAAA	PCR primers for amplification of the upstream fragment of *FgILV2*.
A3<br>A4	ATggatccAGGGTAGAGTCGATTGAAGCG<br>ATgagctcAAATGGGGCGCAGTTAAAAC	PCR primers for amplification of the downstream fragment *of FgILV2.*
A5<br>A6	AGCCCTGCCTTCAACTTGGA<br>TCTGGTATGGATTTACGCCA	PCR primers for the identification of *FgILV2*disruption mutants.
B1<br>B2	ATggtaccCGGTTGTCGATACGATTCCA<br>ATctcgagAAAATCAATGGGCCATGGAG	PCR primers for amplification of the upstream fragment of *FgILV6*.
B3<br>B4	ATggatccTCCCGGTTAAATCAGATACCT<br>ATgagctcTCATCCTATGTTTCTGGGCA	PCR primers for amplification of the downstream fragment *of FgILV6.*
B5<br>B6	CGGGCAATGTTGACCAACT<br>TGATTACTGCACGGGATGAA	PCR primers for the identification of *FgILV6*disruption mutants.
ilv2-com-F<br>ilv2-com-R	ATggatccGGTTTATGGCATTTTGGTCAG<br>ATctgcagAAAGAGAAAGCTCTCAAGCGA	PCR primers for amplification of the entire *FgILV2* gene including 579-bp the promoter region and 530-bp terminator region.
ilv6-com-F<br>Ilv6-com-R	ATggatccCGGTTGTCGATACGATTCCA<br>ATctgcagTACTCTGGCCAAGCACTCAA	PCR primers for amplification of the entire *FgILV6* gene including 1135-bp the promoter region and 863-bp terminator region.
Ilv2-all-F<br>Ilv2-all-R	ATGCTCCGAAGTCGCCCTA<br>TCAAGAACCGTGAAGACCACT	PCR primers for amplification of full cDNA sequence of *FgILV2*.
Ilv6-all-F<br>Ilv6-all-R	ATGGCATCTCTGCGGTCGTT<br>TTAACCGGGAGGGAGCTG	PCR primers for amplification of full cDNA sequence of *FgILV6*.
Ilv2-RT-F<br>Ilv2-RT-R	CCGTGGTACCATTGTCATGAA<br>TTCTCCTCAGCCTTGCCAGT	PCR primers for the detection of *FgILV2* transcription.
Ilv6-RT-F<br>Ilv6-RT-R	AGGACTTCCACCCTAGCAAGT<br>ATCAACAACATCATCCGCCT	PCR primers for the detection of *FgILV6* transcription.
PKS12-RT-F<br>PKS12-RT-R	TGGTGTAGATGCTGTTCGTGT<br>TGAACTTTTCGAGGACGGAT	PCR primers for analysis of *PKS12* expression.

AurJ-RT-F<br>AurJ-RT-R	AAAAAGCAGCCAAGGAGCAT<br>TTCTGATGACACGCTCCCGTA	PCR primers for analysis of *AurJ* expression.
Gip1-RT-F<br>Gip1-RT-R	TGCGGTATCAGGTCACAAA<br>ATCAAAGTCTCCCACCGTGAA	PCR primers for analysis of *Gip1* expression.
Gip2-RT-F<br>Gip2-RT-R	CACCAGCCCTACACCATCTAA<br>TTTCCAAAGCGAGAAACAGC	PCR primers for analysis of *Gip2* expression.
AurF-RT-F<br>AurF-RT-R	ATCTTCAGTCTTGACCATCCC<br>TACCCAAGATGTTCTGGCAA	PCR primers for analysis of *AurF* expression.
AurR2-RT-F<br>AurR2-RT-R	AGGTCGTTGACACGGCAT<br>TGTGCCAGGAGTAAACTTTGA	PCR primers for analysis of *AurR2* expression.
Fgactin-F<br>Fgactin-R	ATCCACGTCACCACTTTCAA<br>TGCTTGGAGATCCACATTTG	PCR primers for amplification of the reference gene actin, in real-time PCR.
Neo-F<br>Neo-R	GGAGGTCAACACATCAATGCT<br>TCAGAAGAACTCGTCAAGAAG	PCR primers for amplification of the G418 resistance gene.
FgF<br>FgR	TATCCCTTATGGGTCTTGGT<br>GGACCGTAAACTTCTTCTGCA	PCR primer pair for specific detection of *F. graminearum*, in real-time PCR.
